# How patients perceive the therapeutic communications skills of their general practitioners, and how that perception affects adherence: use of the TCom-skill GP scale in a specific geographical area

**DOI:** 10.1186/1472-6963-8-244

**Published:** 2008-12-01

**Authors:** Michèle Baumann, Cédric Baumann, Etienne Le Bihan, Nearkasen Chau

**Affiliations:** 1 INtegrative research unit on Social and Individual DEvelopment (INSIDE), University of Luxembourg, Luxembourg; 2 EA4003, Nancy Université, Ecole de Santé Publique, Nancy, France; 3 INSERM, U669, Paris, F-75014, France; 4 Univ Paris-Sud and Univ Paris Descartes, UMR-S0669, Paris, France

## Abstract

**Background:**

To study: (1) the structure and test-retest reliability of a measure of how patients perceive the therapeutic communications skills of their general practitioners (TCom-skill GP), and (2) the associations of that scale with socio-demographic and health-related characteristics, and adherence.

**Methods:**

A total of 393 people who lived in the same geographic area and invited to attend a preventive medical centre for a check up were asked to complete a self-administered questionnaire concerning TCom-skill GP (15 items), socio-demographic and health-related characteristics, and to answer two questions on perceived adherence.

**Results:**

The average age of respondents was 46.8 years (SD 14), and 50.4% were men. The TCom-skill GP score was one-dimensional, had high internal coherence (Cronbach α 0.92), and good test-retest reliability (intra-class correlation coefficient 0.74). The overall score was positively related to increasing age. Respondents aged 60+ were more likely to be adherent. The higher the score, the higher the probability of adherence. Multivariate analysis showed that the TCom-skill score was associated with advancing age and the number of consultations with the GP during the previous 3 months, but not with gender, living alone, being employed, job category or educational level. Multivariate analysis also showed that adherence was associated with TCom-skill GP score which concealed the association between adherence and advancing age observed in univariate analysis.

**Conclusion:**

The TCom-skill GP scale probably has value in assessing the quality of doctor-patient relationships and therapeutic communications. The psychometric properties of the TCom-skill GP scale were appropriate for its use in this context. Adherence related to the TCom-skill GP and the latter related to the age of patients and the number of their previous consultations. The TCom-skill GP scale may be a useful way to assess, in a specific geographical location, the impact of medical professional training on therapeutic communication.

## Background

The relationship that a patient has with his/her general practitioner (GP) is well known to affect the information they exchange. If Balint group participation helps the GPs to be more patient-centred [1] is beneficial to their working life as physicians (competence, professional identity, a sense of security, job satisfaction and preventing burnout) [2] the patients' characteristics thought to influence patient-GP relationship include age, marital status, educational level, socio-occupational category, level of income, race, ethnic background, and language [3,4]. Degree of communication achieved, amount and quality of the information provided, feelings of partnership, respect of the physician for the patient, and the ability of the physician to motivate the patient are of paramount importance. Such factors have been investigated in terms of their impact on both general satisfaction [5-9] and subsequent adherence [5-7,10]. Patients are much more likely to be satisfied with care when they establish a rapport with the physician, are given/retain information about their symptoms and the treatments prescribed, are able to ask questions and to discuss their ideas and those of the healthcare provider, and perceive the physician as seeking to build a partnership [5]. Not surprisingly, major inter-personal shortcomings such as failure to communicate or establish a care provider-patient partnership at all may have a deleterious effect on adherence [11].

Patient non-compliance is an important problem of public health which is likely to increase in successive years as the population ages [12]. During the last decades biomedical approaches have been used to investigate more than two hundred factors with the potential to predict adherence with treatment [12,13]. Assessments of concordance (negotiation between patient and healthcare provider concerning suitable treatment and its implementation) and shared decision-making, have emphasized the role of reciprocity/mutuality [14,15], and the cognitive and emotional dimensions of the physician-patient relationship reflect a working alliance strongly associated with patient adherence to and satisfaction with treatment [16]. Unfortunately, the literature indicates that physicians tend to see non-adherence as due to lack of cooperation by patients [17] and that most caregivers believe they provide sufficient information and explanation for a patient to fully comply with their instructions, and see non-adherence as an irrational response on the patient's part. Some studies have shown that providers' attitudes, their willingness to listen to patients' concerns, to take account of their preferences and to give them appropriate information also play important roles [7,18]. A good doctor-patient relationship is that perceived as good by both the doctor and, more importantly, the patient. Adherence is more likely when physicians give explicit and complete instructions, when patient and physician feel positive about each other, and when patients have confidence in physicians and are satisfied with the care they are receiving [18]. In short, when patient's requirements and expectations are met, the quality of the doctor-patient relationship improves, as does the level of patient satisfaction with the care he/she receives, leading to improved adherence to medical advice and fewer consultations [19].

Recently, a meta-review of adherence intervention studies was conducted, in which corresponding authors were invited to join the International Expert Forum on Patient Adherence and to participate in a web-based focus group discussion. The development of simple interventions has the most potential to foster patient adherence, preferably within a multidisciplinary setting including patient input. Elucidation of patient perspectives requires open communication about their expectations, needs and experiences in taking medication and about what might help them to become and remain adherent [20]. A question of interest is whether the perceived therapeutic communications skills of GPs is influenced by socio-demographic and health-related characteristics of patients as stated by certain studies [7]. Such knowledge is important when conducting interventions to improve the patients' perceived therapeutic communications skills of GPs.

In order to study patient perceptions of the therapeutic communication skills of GPs we require a measure with relevant psychometric properties. To our knowledge, there are no published scales of the perceived therapeutic communications skills of GPs, most studies have focused on satisfaction. It should be noted that a patient may regard his or her GP as providing a satisfactory service despite a lack of certain skills. For example, a patient may report that the quality of the information received is satisfactory but that there is not enough presents during the consultations [5]. There is thus a need to explore the feasibility of using a broader definition of the appropriateness of prescribing in general practice by developing tailor-made measures and exploring their influence on patient outcomes. To that end, we conducted focus groups and individual interviews involving 40 health-care users, 21 general practitioners, and 22 pharmacists operating in north-eastern France. Qualitative analysis of the data identified 15 generic criteria describing therapeutic communication skills involved in deciding on treatment and patient follow-up [21]. The TCom-skill GP score represents determinants of the quality of the interpersonal doctor-patient relationship and therapeutic education [10,19].

Our aim was to determine the level of competence of GPs with regard to therapeutic communications as perceived by patients, and its effect on adherence. The specific objectives were to: 1) study the structure, and test-retest reliability of a measure of patient perceptions of the therapeutic communication skills of general practitioners; and 2) analyse the associations of the TCom-skill GP scale with socio-demographic and health-related characteristics, and adherence.

## Methods

### Sample

Subjects who lived in the same geographic area were recruited over a two-month period at a preventive medical centre in north-eastern France. The French State Health Insurance Office offers a regular free medical check-up to all working people aged 18–70 years. This approach was adopted for practical reasons. It should be noted that the therapeutic communications skills evaluated were those of the patient's usual GP, not of the doctor who conducted the check-up.

### Ethics

The protocol was approved by the supervisor of the National Health Insurance Fund for Social Workers – CNAMTS Paris, France. All subjects gave written consent prior to participation.

### Data

Data remained anonymous and were collected by research assistants who had no connection with the health centre. Respondents completed a self-administered questionnaire comprising 15 items relating to TCom-skill GP (Table 1) with responses ranging from 0 = never to 9 = always (the English version was translated, and back translated). Two validated questions concerned perceived global adherence [22,23]: (a) "Do you take the doses prescribed by your GP?" and (b) "Do you take your medicine at the time recommended by your GP?" (responses ranged from 0 = never to 9 = always). Five socio-demographic characteristics were recorded: age, sex, living alone (yes/no), current employment (yes/no), level of education (lower than high school diploma/high school diploma or higher). Number of consultations with a GP over the previous 3 months (0, 1, ≥ 2) was also determined.

**Table 1:** General practitioner therapeutic communications skills

**My general practitioner...^1^**	**No. of subjects**	**Mean (SD^2^)**	**[Min-max]**	**Missing values (%)**	**Lowest Response (%)**	**Highest Response (%)**	**Factor 1 (1^st ^eigen value 7.5)**
	
1. Takes time to listen to me	391	7.14 (2.03)	[1 – 9]	0.5	0.0	39.1	0.74
2. Does everything to make me feel I can trust him/her	388	6.82 (2.08)	[0 – 9]	1.3	0.5	31.9	0.76
3. Explains what the treatment is for	388	7.10 (2.17)	[0 – 9]	1.3	0.5	40.5	0.75
4. Takes account of my preferences in prescribing medication	382	6.54 (2.66)	[0 – 9]	2.8	4.7	35.1	0.57
5. Gives me the impression he/she has respect for me	387	7.67 (1.79)	[0 – 9]	1.5	0.8	49.1	0.78
6. Gives me information on the side effects of medication	387	5.76 (2.87)	[0 – 9]	1.5	7.0	25.3	0.68
7. Emphasises which are the most important drugs	384	6.66 (2.44)	[0 – 9]	2.3	1.6	32.0	0.75
8. Discusses any difficulties I have in complying with the treatment	370	5.42 (3.04)	[0 – 9]	5.8	11.6	24.1	0.67
9. Explains things in simple words	388	7.07 (2.15)	[0 – 9]	1.3	1.6	36.9	0.70
10. Offers new treatment	373	5.21 (2.74)	[0 – 9]	5.0	8.6	14.8	0.55
11. Writes the prescription legibly	390	4.83 (3.31)	[0 – 9]	0.8	14.1	25.9	0.48
12. Lets me ask questions	387	7.44 (2.00)	[0 – 9]	1.5	0.3	47.3	0.72
13. Gives me incentives to comply with the treatment	382	6.34 (2.53)	[0 – 9]	2.8	3.1	29.1	0.71
14. Gives me advice on prevention (diet, physical activity)	384	5.84 (2.76)	[0 – 9]	2.3	6.5	21.7	0.67
15. Gives the impression he/she knows his/her job	388	7.53 (1.78)	[0 – 9]	1.3	0.5	42.5	0.68

^1 ^Scale of items: 0 = never to 9 = always.

^2 ^SD: Standard deviation.

### Statistical analysis

Age was categorised in four classes: ≤ 39, 40–49, 50–59, and ≥ 60 years. The magnitude of the TCom-skill GP was defined as the sum of responses to the 15 items, which was then standardised from 0 to 100 (the higher the score, the greater the respondent's approval).

The TCom-skill GP was studied by examining the distribution of responses to its items. Its one-dimensionality was assessed using principal component analysis; its internal consistency reliability using the Cronbach α coefficient; and its test-retest reliability using the intra-class correlation coefficient (ICC, based on an analysis of variance model assuming a simple random effect on the 86 subjects who participate to both stages) [24].

We assessed the relationships between the TCom-skill GP score and various socio-demographic and health-related factors by using first one-way analysis of variance, then a multiple linear regression model to take into account all those factors simultaneously.

Adherence was dichotomized because responses to the two questions about perceived adherence were highly correlated and the distribution of the responses was very skewed (most respondents answered "always" to both): respondents who replied "always" to both questions were considered adherent and all the others were recorded as non-adherent. We assessed the relationships of adherence on one side with TCom-skill GP score, and with socio-demographic and health-related factors on the other, using first simple logistic regression models then a multiple logistic regression model to take into account all factors or covariates. sAnalyses were performed using statistical software SAS v8.1 and R v2.7.

## Results

The characteristics of the 393 subjects are shown in Table 2. Their average age was 47 years (SD 14), and 50.4% were male. Three quarters of the sample had consulted a GP within the previous 3 months. The mean TCom-skill GP score was 72.3 (SD 18.7). Fewer than half of the respondents (44.9%) reported being adherent.

**Table 2:** Characteristics of sample (n = 393)

	%
	
**Age**	
≤ 39 years	32.7
40–49	23.0
50–59	23.8
≥ 60	20.4
	
**Mean (SD), years**	47.0 (14.0)
	
**Male**	50.4
	
**Living alone**	15.4
	
**Employed**	56.8
	
**Socio-occupational category**	
Managers and intermediate professionals	50.4
Clerical workers	25.5
Manual workers	14.8
People who never worked	9.4
**Level of education (high school diploma or higher)**	51.5
	
**Number of consultations with the GP during the previous 3 months**	
None	25.8
1	39.1
2 or more	35.1
**TCom-skill GP score [0–100]**	
Mean (SD)	72.3 (18.6)
**Adherence**	45.0

### Internal validity and reliability of the TCom-skill GP scale

Table 1 shows that completion of all the items of the scale was very good. In total, the responses to the items reaching the maximum/minimum values (0 or 9) were always lower than 50%. With the exception of items 8 and 11, the percentage of "never" responses was lower than 10%, and the proportion of "always" responses was more than 30% for nine items. Principal component analysis showed that the TCom-skill GP scale was one-dimensional, thus validating calculation of a score. The first factor explained 49.8% of the variance which was markedly higher than those for the second (8.8%) and third (1.2%) factors. The Cronbach α coefficient was 0.92. The correlation coefficient between every item and the TCom-skill GP score was higher than 0.55.

In order to assess the test-retest reliability of the TCom-skill GP scale, subjects were mailed the same questionnaire 15 days later. Of the 393 subjects, 86 returned the questionnaire. The intra-class correlation coefficient of the TCom-skill GP score was 0.74 [0.66–0.82]. It should be noted that the subjects who completed the second measure did not significantly differ from the others according to age, gender, education, living alone, being employed, socio-occupational category or number of consultations with GP.

### Relationships between the TCom-skill GP scale and socio-demographic characteristics and health-related factors

Table 3 shows that univariate analysis failed to find an association between the TCom-skill GP score and sex, living alone, being employed, or socio-occupational category. These factors also failed to reach significance in the multiple regression model. Educational level was significantly linked with the TCom-skill GP score in univariate analysis but did not reach the significance level of 0.05 in the multiple regression model, suggesting that the association was accounted for by age and number of consultations with a GP during the previous 3 months. Advancing age and the number of consultations with the GP during the previous 3 months were strongly associated with higher TCom-skill GP score.

**Table 3:** Relationships between TCom-Skill GP score and various factors (n = 393)

	**One way anova**			**Multiple regression**		
	**β **	**(se)**	**p**	**β **	**(se)**	**p**
**Age **			<0.0001			0.0005
≤ 39 years	-13.9	(2.5)		-12.8	(3.3)	
40–49	-7.4	(2.7)		-8.0	(3.4)	
50–59	-2.2	(2.7)		-3.8	(3.2)	
≥ 60	0	-		0	-	
**Sex**			0.3018			0.0844
Male	-2.0	(1.9)		-3.4	(2.0)	
Female	0	-		0	-	
**Living alone**			0.3153			0.2184
Yes	2.7	(2.7)		3.3	(2.7)	
No	0	-		0	-	
**Current employment**			0.1190			0.1640
Yes	-3.0	(1.9)		3.3	(2.4)	
No	0	-		0	-	
**Socio-occupational category**			0.8893			0.9014
Managers and intermediate professionals	1.7	(3.4)		1.4	(3.9)	
Clerical workers	1.1	(3.7)		-0.4	(3.9)	
Manual workers	-0.3	(4.0)		0.6	(4.4)	
People who never worked	0	-		0	-	
**High school diploma or higher**			0.0318			0.2915
Yes	-4.1	(1.9)		-2.4	(2.3)	
No	0	-		0	-	
**Number of consultations with the GP during the previous 3 months**			0.0022			0.0228
None	-8.5	(2.5)		-6.4	(2.5)	
1	-4.9	(2.2)		-4.9	(2.2)	
2 or more	0	-		0	-	

### Relationships between adherence and the TCom-skill GP scale, socio-demographic characteristics and health-related factors

Table 4 shows that the higher the TCom-skill score, the higher the probability of being adherent. That result holds whether the other variables were taken into account or not, particularly age. Univariate analysis shows that older people were more likely to be adherent, but the effect was no longer significant when the TCom-skill score was taken into account.

**Table 4:** Relationships between adherence and TCom-skill GP score and various factors (n = 393)

	**Simple logistic regression**			**Multiple logistic regression**		
	**β**	**(se)**	**p**	**β **	**(se)**	**p**
**TCom-skill GP**	0.033	(0.006)	<0.0001	0.027	(0.007)	<0.0001
**Age **			0.0002			0.1164
≤ 39 years	-1.30	(0.31)		-0.91	(0.41)	
40–49	-1.02	(0.32)		-0.88	(0.42)	
50–59	-0.85	(0.32)		-0.79	(0.39)	
≥ 60	0	-		0	-	
**Sex**			0.0929			0.4672
Male	-0.35	(0.21)		-0.18	(0.24)	
Female	0	-		0	-	
**Living alone**			0.9786			0.5591
Yes	-0.01	(0.29)		-0.19	(0.33)	
No	0	-		0	-	
**Current employment**			0.0715			0.7301
Yes	-0.38	(0.21)		0.10	(0.29)	
No	0	-		0	-	
**Socio-occupational category**			0.7323			0.8671
Managers and intermediate professionals	-0.30	(0.36)		-0.21	(0.48)	
Clerical workers	-0.08	(0.39)		0.02	(0.48)	
Manual workers	-0.29	(0.43)		-0.17	(0.54)	
People who never worked	0	-		0	-	
**High school diploma or higher**			0.1018			0.9589
Yes	-0.34	(0.21)		-0.01	(0.28)	
No	0	-		0	-	
**Number of consultations with the GP during the previous 3 months**			0.4449			0.9538
None	-0.34	(0.27)		0.09	(0.31)	
1	-0.09	(0.24)		0.01	(0.27)	
2 or more	0	-		0	-	

No association was found between adherence and sex, living alone, being employed, socio-occupational category, educational level or the number of consultations with a GP during the previous 3 months in either univariate analysis or multiple logistic model.

## Discussion

The present study shows that the TCom-skill GP scale of patients' perceptions has an appropriate structure and is reliable. The TCom-skill GP scale probably has value in assessing the level of professional competences of GPs with regard to therapeutic communication perceived by patients, and its effect on adherence. We see the link between adherence and TCom-skill like a confirmation of the external validity of the scale. Adherence related to the TCom-skill GP and the latter related to the age of patients and the number of their previous consultations. The TCom-skill GP scale may be a useful way to assess, in a specific geographical location, the impact of medical training. We suggest also to use the groups of patients to grade each GP, and recommend the advantages of adjusting for age and number of consultations when evaluating GPs.

The therapeutic communication represents a component of the quality of the interpersonal doctor-patient relationship [10,19,21] and can be used to evaluate professional skills that address the needs of healthcare system users [18]. The TCom-skill GP scale was positively related to age and the number of consultations with the GP during the previous 3 months. Respondents more than 60 years old were more likely to be adherent. The proportion of adherent patients in this study was 45%, a similar figure to that reported elsewhere (50% to 70%) regardless of the disease, the prognosis, the setting, or the measure used [17].

The result concerning age is in accord with findings of other authors [25]. Throughout life, people are subject to social, cultural and psychological influences, and cannot be considered simply as passive recipients of a prescribed medication. Adherence as a behaviour may reflect a complex interaction between age and reaction to communication with the GP. Older individuals are more likely to have experienced serious health problems, and fear of disease and complications tends to increase adherence. Some authors note poor adherence among elderly people, perhaps due to reduced physical capacity, polypharmacy, or problems with memory or comprehension [26]. Older people are likely to have had the same GP for a longer time, and to consult him or her more often because of their health status. They are more likely to be affected by disability and malnutrition, and are particularly prone to polypathology and to needing ongoing treatment [27,28]. It should be noted that our study highlighted a lower level of TCom-skill GP and perceived adherence among younger age groups. Issues in this area include finding better ways to talk to patients, especially for younger people, developing new approaches to explaining the treatment options, and facilitating transfer of information. Patients look to practitioners for teaching.

Our study failed to find an association between TCom-skill GP and living alone, being employed, educational level or the patient's job category. These results are important because they suggest that these social and economic determinants do not influence the patient's perceptions of TCom-skill GP or, consequently, access to health care provided by the GP [29-31].

### Limitations of the study

Limitations of the present findings relate to sample recruitment and measurement of adherence. First the cross-sectional design used precludes any formal conclusion about the causality of the associations between TCom-skill GP and their correlates and between adherence and their determinants. Nevertheless, in the case of sociodemographic correlates, and especially gender, job category, such limitation is a minor one. Second, respondents were volunteers concerned about their health, and consequently not fully representative of the general population (farmers, farm workmen, craftsmen and tradesmen are not seen by the preventive medical centres). Interpretation of the data therefore requires caution. Third, the data were self-reported and not objectively measured. However, the self-administered health history questionnaire is considered reliable and valid [32]. Measurement of adherence is a delicate process. Indeed, the diversity of approaches to measurement is the main obstacle to research in this area. Each strategy has its own advantages and limitations regarding implementation and the interpretation of results, all of which affect reliability [15,33].

The method adopted here – subject report – is the most widely used. It is indirect and often presented in the literature as a simple, reliable tool that is easy to implement. The most common criticism is that subjects tend to overestimate their degree of adherence, either to please the interviewer or because they are afraid of his or her disapproval [34]. In order to counteract interviewer bias here, the two questions on perceived adherent behaviour were self-administered after the subjects had been assured of the confidential nature of the investigation. This form of inquiry is sensitive, but it lacks specificity [35]. On one hand, people who report not always taking medication according to their GP's instructions are often telling the truth, but, on the other, some non-adherent patients claim to be adherent. For that reason, only those subjects who stated that they always took correct doses at the correct time were considered adherent here.

### Implications

Although this study does not tell us whether patient expectations and requirements vary according to age, it does raise the issue and certainly suggests that there is a need for further research in the field. The findings do not indicate that the healthcare provider-patient relationship is the only factor governing non-adherence, but do confirm that the mechanism underlying adherence is a complex, multi-factorial phenomenon in which the healthcare provider-patient relationship seems to play a considerable part. Greater understanding of knowledge and beliefs among the public, and improved awareness of age-related variations in patients' behaviour [36], are essential if new preventative strategies are to be developed, and the best results will be achieved if public health programmes are carried out alongside inquiries of this kind. Interventions may need to focus on healthcare provider-patient interactions [7,37].

All public health education and prevention programmes should emphasise the importance of following medical advice. Healthcare providers as well as patients need education in order to improve adherence to treatment [38]. Communication skill is crucial in some exchanges. Entrants to the medical profession must be given the tools they need to deal with an increasingly demanding patient population. With assistance in the form of information and initial and ongoing training, the skills of GPs can be improved and modified to take into account the changing needs of patients in various social categories. It is important to assess the relationships between the patient's perception of TCom-skill GP, and socio-demographic characteristics with an important effect on access to healthcare, and consequently on social inequality in health [29,30,39].

Therapeutic communication skill of the GP depends not only on the knowledge a practitioner has, but also, to an even greater extent, on his/her ability to make use of that knowledge and explain it to patients. Satisfaction also depends on the quality of the interpersonal relationships that the healthcare provider and his/her patients establish [18]. Practitioners are ethically and legally obliged to pass on information by conducting a dialogue with their patients [7]. However, the education they provide appears to be relatively neglected due to lack of skill, shortage of time, and poor recognition of its importance [7,40]. Our study shows that proper attention should be paid to younger patients and people who have a low level of education or only occasionally consult their GP.

No relationship was revealed between the TCom-skill GP score and socio-economic characteristics of the patients. This is important given that consultations with most specialists are preceded by contact with the GP, who had an important role in work-related health, liaising between workers and occupational physicians, other care providers, employers, and the compensation systems in France [31]. However, our finding needs to be confirmed by studies in other populations.

## Conclusion

The present study takes into account the criteria that determine the quality of the interpersonal doctor-patient relationship. Changes of the kind described above could markedly improve the therapeutic process. The question now is: are practitioners ready to acknowledge the need to reconsider and modify the care they provide for patients? It has never been easier to assess how far we have come and what remains still to be done to ensure that the relationship between patient and practitioner is genuinely open, allowing each party to have a real and positive influence on the other [7,9].

"Concordance" [35] encompasses all aspects of the patient-healthcare provider interaction and thus clearly covers TCom-skill GP. Mutuality can be obtained by patients *and *doctors, and requires the active participation of the latter. This model of interaction could serve as an example of such sharing and patient empowerment [18] – the healthcare provider and patient working together as a team rather than the patient simply following the healthcare provider's orders more or less automatically [7]. This approach can be seen as a prerequisite for the establishment of genuine communication, with the practitioner becoming a therapeutic educator [41].

## Competing interests

The authors declare that they have no competing interests.

## Authors' contributions

MB: Scientific Director of the research, involved in conceiving the study design and writing the manuscript; CB: participated in the design of the study and data analysis; ELB: conducted the statistical analysis and helped draft the manuscript; NC: participated in the data analysis and helped draft the manuscript. All authors read and approved the final manuscript.

## Pre-publication history

The pre-publication history for this paper can be accessed here:


